# The New Building of the Dental Department of the University of Maryland

**Published:** 1882-06

**Authors:** 


### EDITORIAL, ETC.

*The New Building of the Dental Department of the University of Maryland.*—This structure is rapidly approaching completion, and will be ready for occupancy by the middle of July next. It is constructed of brick with fourteen-inch walls two stories in height and situated on an elevated site in such a position that there is unobstructed light on every side.

The entire building contains *forty-nine* windows independent of the doors; *twenty-five* of which are in the Dental Infirmary, rendering it the most complete and best lighted hall devoted to such a purpose in existence. It is the only Dental Infirmary and Laboratory Building that has ever been constructed for such uses; hence every care has been taken to make it as complete in all its appointments and conveniences, both as regards location, position and arrangement as was possible. The extensive grounds of the University rendered the selection of a site which would be best adapted to such a purpose, a matter of choice only. On the first floor is the extensive Dental Laboratory, the Extracting Room, Impression Room, Wash and Water Closets, Closets for Instruments, etc. The Dental Infirmary occupies the entire second story, in which there is not even a column, being one unbroken hall, sixty feet long by thirty feet wide, and capable of accommodating some fifty or more dental chairs.

In addition to the large amphitheatres and lecture rooms of the Medical Department, in which the lectures to the dental students on Anatomy, Surgery, Materia Medica, Physiology and Chemistry will be delivered, there is a large Lecture Hall devoted to purely dental lectures, containing elevated seats capable of accommodating hundreds of dental students.

The arrangements throughout of this Dental Institution are, beyond doubt, more complete than those of any other in existence; and the prospects for a successful beginning are most encouraging. It is universally acknowledged that the advantages offered dental students by the Dental Department of the University of Maryland far excell any within the power of separate dental schools. And as the University of Maryland was organized in 1806, its dental diploma will bear a date *thirty-four years older* than that of the oldest seperate dental school. Besides, the high reputation which the diploma of the Medical Department of the University has always born, cannot fail to render its dental diploma equally valuable, and assure its recognition in foreign countries where the diplomas of separate dental schools are now denied recognition, as in England and Germany, for example.

It is also safe to predict that in a few years hence, separate dental schools will have become extinct, and dental education be conducted solely within Medical Universities, where it properly belongs, and where it can be more perfectly pursued.

